# Foreign Medico-Psychological Literature

**Published:** 1861-07

**Authors:** 


### FOREIGN MEDICO-PSYCHOLOGICAL LITERATURE.

Our Retrospect of current Medico-Psychological Literature will embrace the following subjects:

1. The Responsibility of Epileptics.
2. Mental Hyperæsthesia.
3. Cysticercus Cellulosæ in the Brain.
4. On a peculiar Lesion of certain Nervous Centres.
5. On the production of Cerebral Diseases by the Atmosphere of Cafés.

### 1.—*The Responsibility of Epileptics.** By M. Baillarger.

The question of the responsibility of epileptics appears to offer several points worthy of interest, and I propose to submit, as MM. Devergie and Tardieu have already done, some considerations regarding this question.

M Trousseau has only spoken in his paper of sudden impulses leading to homicide and suicide, of that which is generally described under the denomination of transitory madness. MM. Devergie and Tardieu have also confined themselves to this transitory madness. I do not wish to enlarge the bounds of this discussion, but there is, however, one point besides transitory madness which appears to me to merit examination. I refer to the mental condition of certain epileptic patients who, without being insane, present, nevertheless, in relation to the intellectual and moral faculties, certain special characteristics which it is impossible not to attach to their malady. These characteristics, although they do not constitute a state of madness, ought not the less to be taken into serious consideration when it concerns us to form a judgment on actions imputed to epileptics.

I commence by that which bears the mark of transitory madness.

M. Trousseau has very clearly stated his opinion on this question.

When a man, says he, commits a murder upon a sudden impulse and without motive, if that man have not previously shown symptoms of madness, and if he were not in a state of intoxication, his action should almost always be explained by the existence of epilepsy.

---

* This discourse was intended to be delivered from the Tribune of the Academy of Medicine at the sitting of 19th of March last, when the discussion on epilepsy and cerebral congestion raised by the paper of M. Trousseau was suddenly brought to a close.

This doctrine, if demonstrated, would assuredly possess great importance in legal medicine.

There are certainly cases of transitory madness very difficult to judge of, and what an assistance it would be to prove that the patient was really suffering from epilepsy.

I will only cite one example.

A vinedresser in the neighbourhood of Lyons was suddenly seized with a fit of shivering. He took up a mattock and killed three of his children who were near him in the house. A hundred steps from there he also killed his wife and his last child. Having accomplished all these murders he went and gave himself up.

This man was not intoxicated; he had never previously evinced signs of madness; there was no apparent motive to explain his action. He came, therefore, within the conditions indicated by M. Trousseau. He ought to be epileptic.

M. Bottex, the physician charged with the examination of the vinedresser, discovered that he had experienced vertigo and giddiness some days before the event. Besides this, he was sad, melancholy, and appeared to have had some ideas of suicide. Other testimony established that he was much attached to his wife and children.

The case then appeared most simple, and it seemed that transitory madness ought to be admitted without difficulty. But here a witness came forward to speak to a strange proposition which the murderer had held ten months before. He had said that a man who should kill his family would get off with a few months' imprisonment, because the physicians would make him out to be mad. In addition to this he had remarked since his arrest that one of his children having survived its mother some hours, became her heir, and that he himself was heir to this child; his wife's property ought, therefore, to come to him.

Need I speak, gentlemen, of the doubts which then arose in the physician's mind? All result became questionable, and in the Report presented to the magistrates, the existence of an access of transitory madness was only presented as a simple probability.

The murderer was condemned to death, but the punishment was commuted for that of penal servitude for life.

Suppose, gentlemen, that in a case so embarrassing one had discovered epileptic vertigo, what light would this fact have thrown on an action apparently so inexplicable?

I can only repeat, then, that the doctrine of M. Trousseau, if it could be demonstrated, would be one of very great importance in legal medicine; but at present this doctrine is without foundation. There are many scientific observations of transitory madness which appear completely foreign to epilepsy. M. Tardieu has cited several which are very remarkable, and I do not think I need insist on a point which has been so well handled.

There exist, then, at least, two species of transitory madness; the one connected with epilepsy, the other totally independent of that malady. It remains to inquire if there are, between these two species, any differential characteristics which would permit of their being distinguished. It will be understood that if these characteristics existed the medical jurist might avail himself of them in certain cases, in order to establish, at least, the probable existence of epilepsy, which it would not otherwise have been possible to discover.

For some years past, laudable efforts have been made to assign special characteristics of epileptic madness. It has been demonstrated that this species of madness has, up to a certain point, its proper physiognomy.

Nevertheless, the data furnished under this head by MM. Aubanel, Delasiauve, Jules Falret, and especially by M. Morel, scarcely seem to me sufficient to be of service in distinguishing the transitory madness of epileptics from the same disease as exhibited in persons not afflicted with epilepsy.

It is known, for instance, that epileptics preserve no recollection of their accesses, and the loss of memory has been indicated as a symptom proper to epileptic madness. This sign might be important, but it cannot possess any utility for the present purpose, because it exists where transitory epileptic madness is wanting, and where the madness is independent of epilepsy.

There are in Marc's work four observations in which the patients had lost the recollection of their access. None of these patients were epileptic. All the four had had an access of transitory madness with homicidal impulses.

On the other hand, you have heard M. Devergie relate a curious observation, borrowed from Moreau (of Tours), and in which the patient preserved the memory of his access, although tainted with epilepsy. Georget cites the fact of an epileptic, who, seized with a sudden fury, attacked every one he met, and killed three persons. Such was the terror he inspired, that it was believed impossible to arrest him without shooting him. Georget adds, that this patient perfectly remembered the murders he had committed.

It does not appear, then, at present, that we ought to account the loss of memory as sufficient to warrant us in referring certain cases of transitory madness to epilepsy.

The best argument which, as it appears to me, we can invoke in favour of the extension which M. Trousseau wishes to give to epilepsy in its relation to transitory madness, is the facility with which nocturnal attacks, and, above all, simple vertigo may pass unperceived.

I have had under my care a lady of the south, who had been married, being epileptic, without any one being aware of it; besides, it is well demonstrated that there are maladies which only have accesses at extremely long intervals; if these attacks come on during the night, the malady may remain completely unknown.

Let us suppose the case of homicidal fury succeeding one of these isolated accesses, how difficult would it be to avoid error in such a case!

The following is an observation of this kind, which appears to me to possess great interest, and which I have no need to recommend to the attention of M. Trousseau.

A councillor of a town in Germany was seized with a sudden fury in the middle of the night; he attempted to kill his wife, and to throw her out of the window. She struggled for half an hour, and the fury of her husband was then appeased. He appeared exhausted by the efforts he had made. Some instants before this access of the fury, the respiration of this patient became stertorous; his wife, alarmed, wished to relieve him, and it was then that he attacked her.

It is a singular thing that Marc, who relates this observation, does not appear to have suspected the existence of epilepsy. The stertorous breathing, followed by fury, did not awaken in his mind any suspicion of an access; he wrote a few pages further, in a chapter on the transitory madness of epileptics, without appearing to dream that this fact belonged to it; and nevertheless is it not infinitely probable that this sudden fury cannot be accounted for otherwise than by a nocturnal attack of epilepsy? One point very important to remark is, that the patient, during fourteen years afterwards, exhibited no other sign of madness.

Here, then, is an attack of epilepsy totally isolated succeeded by homicidal fury. If the murder had been consummated, if the wife of the patient had not been able to reveal the fact, so important, of the stertorous breathing, would not the error have been almost inevitable?

M. Dumesnil, chief physician of the asylum of Quatre-Mares, at Rouen, cites the observation of a soldier who was brought before a court-martial for grave insults towards his superiors, and it was not till long afterwards that he was discovered to be subject to epileptic vertigo.

These facts, and many others, may be quoted to prove that transitory madness is more often connected with epilepsy than has hitherto been supposed.

This is, I believe, the sole conclusion which can be drawn from this discussion, besides the too absolute doctrine of M. Trousseau.

I pass to the second point which I proposed to examine, that is to say, the influence of epilepsy on the intellectual and moral dispositions of certain epileptics not insane, and to the consequences which the medical jurist may draw from them.

The patients of whom I wish to speak present special traits which are remarked by all authors on the subject. "They have," says Esquirol, "exalted ideas; . . . they are very susceptible, irascible, obstinate, difficult to please, capricious, odd; they all possess some peculiarity of character."

M. Calmeil remarks, that all epileptics not yet insane are very irascible, very impressionable, and disposed to false interpretations.

That, says he, which scarcely moves a man of ordinary susceptibility causes to them a feeling of profound trouble.

M. Delasiauve indicates the same traits in the character of certain epileptics; he concludes that this condition ought not to be considered a real disease, but an extra-physiological disposition.

I think I need not make more references. All authors are, in fact, agreed in admitting this fact, that epilepsy, before leading to complete insanity, produces very important modifications in the intellectual and moral condition of certain patients; these sufferers become susceptible, very irritable, and the slightest motives often induce them to commit acts of violence: all their passions acquire extreme energy.

It remains to examine up to what point we ought to pay attention to these special dispositions, when these epileptics are called to answer acts more or less serious.

I am unable to quote the opinion of any jurist upon this point; but besides epileptics, there are other subjects who without being insane present, nevertheless, intellectual and moral dispositions such that one cannot avoid explaining the anomalies which they exhibit otherwise than by a vicious organization.

The following is the opinion of a magistrate who has given great attention to mental diseases, in regard to the latter class of cases.

Whenever, says he, an hereditary germ or a purely native disposition renders the exercise of reason more difficult to a person whom we account of sane mind, what are we to conclude? That the struggle ought to be greater on his part to overcome these organic obstacles which he is always certain to conquer, so long as his personal power subsists, and he is determined to use it. . . . . It is as certain in physiology, as in philosophical jurisprudence, that we ought not to seek for causes modifying free-will in these inequalities of moral and intellectual character. It is of little importance that the cause may be fatal and hereditary; so long as the germ hereditarily transmitted be not developed in a way to engender insanity, the power of the will is sustained, and in this case the individual is accountable for all actions.

Are we to apply to the epileptics of whom I have spoken so absolute a rule?

This would be, as it appears to me, to exhibit excessive severity.

Without doubt these epileptics are not insane; but if the special condition which has been developed in them by disease does not entirely destroy free-will, we may, I think, without danger to society, acknowledge that it does in many cases modify it.

I believe, then, that the duty of a physician, when it concerns him to consider the responsibility of an epileptic, ought to consist often, where insanity does not exist, in making plain and apparent the influence of the disease on the intellectual and moral dispositions of these patients. At times he will have to remark a commencement of weakness of intellect, as was the case with Lecouffe, accused of assassination, whose case was examined by Georget: more often he ought to bring into relief that irritability, those violent passions, that suspicious character, of epileptics, the exaggeration of their sentiments—in a word, all those marks which, without constituting insanity, nevertheless place these patients beyond the common rule.

It will often happen that he may thus obtain for the epileptic, not absolution but mitigated punishment.

Whatever may be the differences in theory, there are facts whose influence it is impossible not to acknowledge. An epileptic not insane commits an attempt to murder; premeditation is perfectly demonstrated; the murderer has designedly provided himself some days before with the knife which he has used. M. Boileau of Castelnau attempted to obtain the acquittal of the patient: in this he did not succeed; but the punishment was mitigated two degrees, and the epileptic, though not insane, was only condemned to six years' imprisonment.

How many similar examples might we not cite of crimes committed by men regarded as reasonable since they have been condemned, but towards whom the penalty has been reduced because of doubts raised as to the plenitude of their reason!

I was once employed to draw up a memorial for a man who had attempted to assassinate a magistrate by striking him three blows with a poignard. Premeditation was well established. The question of insanity was raised, and there was this remarkable fact, that the six physicians called to give successively their opinion on the mental state of the accused, divided themselves as follows:—Two considered him insane; two considered he was not; the two last, after three months' examination, made a long report, but refused to pronounce him either sane or insane.

The magistrates decided that this man was not insane, since they condemned him; but for this attempted assassination, made with premeditation against the person of a magistrate, the punishment was only six years' imprisonment.

To resume: it has appeared to me useful to recal:—

1. That besides declared insanity, there exists with certain epileptics a special moral and intellectual condition.

2. That the medical jurist ought, in many cases, to apply himself to make apparent the principal traits which characterize this condition, to extenuate, at least as much as possible, the responsibility of the sufferer.—*Annales Medico-Psychologiques*, Avril, 1861.

---

2.—*On Mental Hyperæsthesia.* By John Ordronaux, Professor of Medical Jurisprudence.

The following suggestive observations occur in an interesting Lecture delivered by Professor Ordronaux before the Students of Columbia College, New York:—

"I wish now to speak of a condition of mind, often predisposing to hallucinations, of which the authorities make no mention, although it is very common, and sometimes even dangerous in its character. It is an exaltation of, or exaggeration in, the rapidity of mental processes, due to the influence of persistent tension upon the brain. As you will naturally infer, it is the unwelcome attendant upon all active minds when overworked. I shall make no separate allusion, at this time, to the probable influences of narcotics in assisting to produce, or to exaggerate when present, this state of the intellect. As I am making a simple psychological inquiry into a form of disorder, I shall confine myself to the essential causes producing it, and shall not venture upon any physiological disquisition into the remote and correlated sources of its origin. We can all agree upon the fact—whatever we may think of its causes—of the existence of a species of mental disturbance, born primarily of fatigue, exhaustion, or prostration; and which, with your permission, and for want of any other name, I shall call a state of *mental hyperæsthesia.* This is the state of mind in which one finds himself whose mental faculties have been strained to their utmost tension for a great length of time. The result of a long and unabated fixedness of attention upon any one train of thought, is speedily to exhaust the mind; and just in proportion to the degree of volitional effort expended, will there ensue rapidity of exhaustion. The mind, at such a time, although greatly fatigued, is not disposed to quiescence, but continues to oscillate under the reflex influence of its original stimulus.

"This, of itself, is not a condition of ill-health, if it can be speedily removed. So long as the strain does not exceed the recuperative powers of the organ thus overtasked, the shock is not immediately dangerous. But we must remember that this unnatural stimulation of a function exhausts the tone of the organ performing it, in advance of the effects of age. So that, with the mind as with the body, we can preserve it in vigour up to a very late period of life, if we will only use it as not abusing it. And I may state, in passing, a curious illustration of this truth in the fact that, at this time, the statesmanship of England is in the hands of men over seventy years of age: while in this country no man is deemed an available candidate for either judicial or political office who has passed the scriptural limit of human longevity. Now a state of mental hyperæsthesia clearly borders upon abuse of the intellectual powers; nor can we wonder, therefore, at the train of melancholy effects to which it gives rise.

"In this condition of things the brain is inordinately active; its blood-vessels are greatly dilated, its whole substance consequently enlarged. It presses in all directions upon the skull, which seems hardly of a size to contain it; and when this cerebral plethora is continued for weeks and months, who can marvel that men in the very maturity of age, and apparently strong enough to work over their desks for nine hours a day, should suddenly drop paralysed—become victims to hallucination and insanity, or, worse still, fall into apoplexies. It is not asserting too much to say, that if our time-pieces were kept wound up to a similar pitch of tension by constantly turning the key, their mainsprings, although made of steel, would not last a month! Yet this is the mental status of many professional men, particularly in large cities, where the unremitting pressure of business, and the fever of competition, stimulate them to unnatural efforts. Persons often overwork their minds unconsciously, because, through the compensating influences of nature, the external effects of the injury are for awhile concealed, and not until some unmistakeable evidence looms up across the intellectual horizon is the offender made aware of his wrong-doing.

"The majority of professional men toil far into the small hours of night and then retire—to sleep? scarcely any, if at all; but only to think over and over again the duties of the morrow, until a hazy forgetfulness, not deserving the name of slumber, steals over the still occupied brain, and leaves it to finish in dreams the disconnected fragments of daily business. Need we ask what is the consequence of this mode of life when protracted? Everything shows us that Nature's laws are never violated with impunity, and slow-footed Justice, halting and lame though she may be, rarely fails to overtake the retreating criminal. In those individuals who habitually overtask the brain, we shall find manifestations of that form of hallucination which is the offspring of intensified and protracted thought. It is true *hallucinatio studiosa*, and the period at which it develops itself will depend upon certain physical causes, not necessary to be mentioned here. Let it suffice to say, that these hallucinations are generally preceded by inability to sleep *soundly*, and this tendency to insomnolence once established, readily passes into that of *coma vigilans*, a state productive of exquisite irritability. When the brain is long robbed of sleep, it loses both the knowledge of, and the ability to, sleep; so that it requires to be re-educated, as it were, into this aptitude. During this condition of vigil it reacts upon the stomach, and this again upon the brain, so that we now have two foci whence nervous irritability can be radiated and interchanged. The famous Laurence Sterne was once in this condition for several months, and Martin Luther, as the result of his protracted mental labours, was often visited by a hallucination that the Prince of Darkness stood before him, and on one occasion went so far in believing it as to throw his inkstand at him. General Rapp tells us that once, desiring to speak with the Emperor Napoleon, he entered his cabinet unannounced. He found him in so deep a reverie that his entrance was unperceived until he intentionally made a noise. Napoleon then recovered, and pointing to the ceiling said: 'Look up there! Do you not see it? It is my star! It is beaming before you. It has never deserted me! I see it on every great occasion.' Dr. Johnson, too, whose mighty intellect could endure a superhuman amount of labour, was the victim nevertheless of hallucination, and one of the most superstitious men of his time. Rare Ben Jonson was also similarly visited, and Andral, the great anatomist, was pursued for a long time by the image of a child, which he had most critically dissected. Leuret, the philosopher, himself a psychologist, was greatly annoyed by visions which he could not rid himself of. And I have several instances noted among my own observations of similar facts. A friend of mine, who is the president of a bank, and a shrewd financier and economist, is exceedingly annoyed by the presence of a bottle of sarsaparilla, which is always spouting its contents before his eyes. The moment he fixes his attention closely upon any object the bottle disappears, but on releasing the mind from this contemplation the bottle returns. Yet none of these men whom I have mentioned were insane, none would have been disqualified at law either civilly or criminally. On the contrary, every one would pronounce them blessed with strong reason. Theirs were cases of mental dyspepsia. I am inclined to think that, in our country, the very laws of business, of society, of education—in a word, the genius of our institutions—favours, and, I may say, forces us into, a preternatural activity of mind. As slowness and deliberation of action are regarded as marks of mental incapacity, so the premium and the prize are assigned to the opposite extreme, and the man in self-defence is obliged to be 'fast.'" — *American Journal of Insanity*, April, 1861.

---

3.—*On Cysticercus Cellulosæ in the Brain.* By Dr. Snell.

This singular parasite is oftenest found in the muscles and in the connective tissue under the skin; but the serous membranes, the liver, spleen, kidneys, eyes, and lymphatic glands sometimes also serve it as a resting-place. It is rarely found in the brain, but there (while in the other organs, with the exception of the eye, it is usually attended with no ill consequences) it usually proves the cause of the most serious symptoms, and of death itself. Dr. Snell of Hildesheim relates the following case:—A man, æt. 24, living in the country, and engaged during the winter in slaughtering pigs, fell ill, April, 1857, with intermittent fever, accompanied by symptoms of great congestion of the head and chest. Somewhat relieved by large doses of quinine, relapses occurred, and he did not completely recover his health. In the course of the summer of 1857, the patient suffered from periodical cephalalgia, which implicated especially the occiput, and kept increasing in severity. There were also weakness of vision, with enlarged pupils, singing in the ears, vomiting, anxiety, and a sense of want of power in the limbs. His mind was frequently somewhat disturbed, and towards the end of September maniacal excitement was suddenly set up. After remaining in this state of exaltation for two or three days and nights, he slept for twenty-four hours, and awoke with his consciousness clear, but without recollection of the attack. His pain in the head and other symptoms of cerebral disturbance returned, and during the next two months he had frequent returns of the paroxysms of delirium, and by the end of the year his mental disturbance had become continuous, and the paralysis had much increased. The appetite and nutrition, however, remained in a normal state. He was brought to the asylum at Hildesheim on the 27th December, being scarcely able to walk and almost blind. His pulse was 96, and he complained of violent pain in the forehead. After seeming at first to improve somewhat, he died on the 29th. At the autopsy, his brain, which weighed 54oz., was found to contain numerous small cysticerci, which, under the miscrocope, were recognised as *cysticercus cellulosæ.* Five of these were attached to the inner surface of the dura mater; but all the others were met with in the grey substance of the organ, and not only in the cortical substance, but wherever the grey matter was found, as within the ganglia and commissures. The greatest number were found grouped together here and there in the cortical substance of the hemisphere. The optic thalami and corpora striata were also thickly beset with them. In the cerebellum only four were found, and they were entirely absent from the medulla oblongata. The white substance nowhere formed the nisus for the cysticerci, although they sometimes pressed into it when they had reached the limits of the grey substance. The entire number amounted to 400, they being for the most part fully developed, containing a transparent fluid. They had, however, in part degenerated, the fluid being yellow, turbid, or puriform. They were usually of the size of a small pea, but several were less than millet seed. The largest did not exceed the size of a large pea. In all other parts the brain was quite normal, nor did it, even in the vicinity of the cysticerci, exhibit any signs of inflammation or other diseased process. The arachnoid also was quite transparent. Notwithstanding a careful search was made for them, no cysticerci could be detected in the muscles, connective tissue, or elsewhere. Nor was there, indeed, any pathological condition found in any other part of the body : so that the symptoms and cause of death in this case would seem to be solely referable to the mechanical compression exerted by the cysticerci. In this case, it is remarkable that no convulsive movements were observed : and the occasional cessation not only of the symptoms of mental disturbance, but of the paralytic manifestations, is of interest.—*Allgem. Zeitsch. für Psychiatrie*, Band xviii. Heft i.

---

4.—*On an Organic Lesion of certain Nervous Centres, not previously recognised.* By Dr. A. Joire.

The organic changes in the encephalon, which are met with amongst those of the insane who succumb under the influence of paralytic dementia, have of late years been the subject of numerous and important researches. Great difference of opinion nevertheless exists amongst men of science, as to the degree of importance to be attributed to these various lesions in the production of general paralysis. It is well known that this affection was scarcely studied until our own day, otherwise than as a grave complication, and the most grave of all which aggravate insanity. It is only in later times that the facts respecting progressive general paralysis have been observed, as manifested beyond the region of its coincidence with insanity.

This habitual association of two morbid forms has led the authors of these researches to consider the same parts of the encephalic apparatus as being by their lesions the common determining cause.

One who has devoted himself more particularly to the study of general paralysis, M. Calmeil, considered inflammation of the grey substance of the convolutions as the cause both of dementia and paralysis ; he considered this affection to be anatomically a diffuse chronic peri-encephalitis. More recently, others have admitted, as the anatomical characteristic of paralysis, the softening of the middle layer of the cortical substance of the convolutions, both resulting from an inflammatory state. Another opinion maintained by MM. Delhaye and Pinel-Grandchamp, and reproduced more lately by M. Foville, was that the intellectual derangement was connected with changes in the grey substance, and the disorders of movement with lesions of the white substance of the brain. Here we perceive arises the idea of a distinction between the organic seat of the two morbid conditions. But we remark that the cause of the double phenomenon is referred to the brain alone. The researches of modern physiology, however, do not permit us any longer to connect the general disorders of motion manifested in general paralysis with lesions of the cerebral hemispheres.

These organs are acknowledged in our day as the material agents of intellectual and instinctive acts; while the phenomena of sensibility and motion belong to the encephalon and other parts.

Our researches relative to the organic changes proper to general paralysis must therefore be based solely on the experimental data of modern physiology, and especially on the distinctions established by M. Flourens between the respective functions of the different parts of the encephalon; and the cerebral hemispheres should henceforth be considered as beside the question.

Such were my impressions, when, recalling the numerous organic changes verified as belonging to paralytic dementia by persevering microscopic researches during more than thirteen years, my attention was vividly fixed in a special lesion seated beyond the cerebral hemispheres, which for nine months I had never failed to observe in general paralysis, and which I had not met with in paralytic lunatics.

This lesion has its seat on the internal surface of the cerebellar ventricle (fourth ventricle), and consists in the presence of *a seemingly gelatinous transparent layer* of variable thickness, amounting sometimes to a millimetre; *the surface of this layer is covered*, *especially on a level with the anterior and inferior coat of the ventricle*, *with a considerable number of salient papillæ or granulations* exactly analogous to the pimples of the skin manifested under the influence of cold, and designated by the name of goose-flesh.

I have observed that in subjects who succumb at an early period after the appearance of general paralysis, this additional layer is very thin, the granulations of the surface infinitely more numerous and smaller, and their aspect gives the idea of scattered grains of sand, or, better still, the particular alteration of the palpebral mucous membrane indicated under the name of granulations of the conjunctiva. This lesion, which I have called *granulations of the walls of the fourth ventricle*, is constant in general paralysis, and has no other seat.

I have carefully observed, in my microscopic researches, the walls of the lateral ventricles and those of the third ventricle; I have not met with anything there of a similar nature.

I do not propose to raise here the important questions which might be evolved in the study of the relations between the lesion I have just pointed out, and the symptomatic manifestations which I attach to it; neither do I propose to examine whether this lesion may be manifested in the encephalon separately, or whether it is connected with the previous existence of a more general alteration of the brain, whence may depend the disorder of the intellect: a complete work embracing the study of these various questions, accompanied by observations made with reference to this subject, will shortly be communicated to the Academy.

My object at this moment is to point out an organic change peculiar to general paralysis to which, up to the present time, attention has not been directed, and to solicit of my alienist colleagues researches which I feel sure will prove confirmatory of my assertions.—(*Annales Medico-Psychologiques*, Avril, 1861.)

---

5.—*On the Insalubrity of the Atmosphere of Cafés and its Influence upon the Development of Cerebral Maladies.* By Dr. Legrand du Saulle.

Dr. L. du Saulle has arrived at the following conclusions, as the result of certain researches on this subject:—

1. Cafés such as they are at present arranged are far from being sufficiently ventilated, and for this reason are unhealthy resorts.

2. Amongst a great number of those who are assiduous frequenters of cafés, we may observe, after a time which it is extremely difficult to define, a sort of special intoxication; peculiar troubles disturb the economy, and at length a marked tendency to cerebral congestion manifests itself.

3. The accidents to which allusion is made are in no way dependent upon alcoholism; they differ from it in much. They are besides met with in sober men, who make the estaminet a place of meeting for business or pleasure, and not a place for drunkenness.

4. What tends to prove the special character of this variety of congestive poisoning, is that all the phenomena, especially in the first and second stages, disappear spontaneously shortly after the cessation of the cause.

5. All the acute or chronic maladies which affect the brain, and the etiology of which remains impenetrable, may about once in ten cases have no other cause than a daily sojourn for a number of years, for one or more hours a day, in the hot and vitiated atmosphere of cafés.

6. The general paralysis of the insane commencing most frequently in congestion, and the atmosphere of cafés conducting often in the long run to this primordial phenomenon, there is reason to ask if this circumstance might not explain, up to a certain point, the very great frequency of general paralysis in men and its rarity in women.

